# Neck and shoulder pain among elementary school students: prevalence and its risk factors

**DOI:** 10.1186/s12889-019-7706-0

**Published:** 2019-10-16

**Authors:** Elham Gheysvandi, Iman Dianat, Rashid Heidarimoghadam, Leili Tapak, Akram Karimi-Shahanjarini, Forouzan Rezapur-Shahkolai

**Affiliations:** 1 Department of Public Health, School of Public Health, Hamadan University of Medical Sciences, Hamadan, Iran; 2 Department of Occupational Health and Ergonomics, Faculty of Health, Tabriz University of Medical Sciences, Tabriz, Iran; 3 Department of Ergonomics, School of Public Health, Hamadan University of Medical Sciences, Hamadan, Iran; 4 Research Center for Health Sciences, Hamadan University of Medical Sciences, Hamadan, Iran; 5 Department of Biostatistics, School of Public Health Sciences Research Center, Hamadan University of Medical Sciences, Hamadan, Iran; 6 Modeling of Non-communicable diseases research center, Hamadan University of Medical Sciences, Hamadan, Iran; 7 Social Determinants of Health Research Center, Hamadan University of Medical Sciences, Hamadan, Iran

**Keywords:** Musculoskeletal complaints, Children, Adolescent, Physical risk factors, Psychological risk factors; Posture.

## Abstract

**Background:**

Neck and shoulder pain is relatively common among children and teenagers and has a negative impact on their physical and psychological health. This study was carried out to assess the prevalence of neck and shoulder pain among elementary school students, and to investigate the relationship between this pain and its risk factors.

**Methods:**

In this cross-sectional study, 693 elementary school students aged 7 to 12 years from Hamadan city, located in western Iran, were selected through the multistage cluster random sampling method. Data were collected through interviews and questionnaires. For the social and psychological variables, the parent version of the Strengths and Difficulties Questionnaire (SDQ) was used. For assessing each student’s posture, an observational checklist, the Rapid Upper Limbs Assessment (RULA), was used. The data was analyzed using the unadjusted (univariate) and adjusted (multivariate) logistic regression.

**Results:**

The prevalence of the neck pain was slightly higher than that of shoulder pain. The prevalence reported over a month was 35.8 and 30.9% for neck and shoulder pain, respectively. The logistic regression analyses showed that, very high desk height (odds ratio (OR) =1.96, 95% confidence interval CI: 1.02–3.74), backward seat pan inclination (OR = 2.10, 95% CI: 1.37–3.24), forward seat pan inclination (OR = 3.12, 95% CI:1.46–6.68), difficulty in viewing the board (OR = 2.54, 95% CI: 1.10–5.84), too much homework (OR = 2.59, 95% CI: 1.49–4.51), RULA score at level III (OR = 2.88, 95% CI:1.64–5.05), and RULA score at level IV (OR = 3.12, 95% CI: 1.72–5.63) increased the risk of neck pain independently. On the other hand, sitting on desk and seat (OR = 0.59, 95% CI: 0.39–0.91) and laying position for doing homework (OR = 0.53, 95% CI: 0.34–0.81) reduced the related risk. Very short desk height (OR = 2.41, 95% CI: 1.26–4.61) and too much homework (OR = 1.94, 95% CI: 1.10–3.42) increased the risk of shoulder pain.

**Conclusion:**

The elementary school students reported a high prevalence of shoulder and neck pain. This study found that improper sitting positions, as well as physical factors such as the school furniture, too much homework, and difficulty in viewing the classroom board, were associated with pain. Proper interventions considering the risk factors assessed in this study, are suggested.

## Background

Neck and shoulder pain is a relatively mild musculoskeletal condition [1], but in recent years it has become a major health problem, and has imposed a heavy burden on the person and community [2–4]. The World Health Organization (WHO) has ranked neck pain and other musculoskeletal diseases as the fourth and tenth health problems, respectively, for years lived with disability [5]. The Data show that, the prevalence of neck pain in the general population ranges from 0.4 to 86.8% in the world [6]. In addition, the Global Burden of Disease Study showed that neck pain is one of the main causes of years lived with disability among adolescents aged 15 to 19. Compared to the other health problems such as asthma, alcohol and drug abuse it has a higher prevalence [7]. Also some studies indicate that shoulder and neck pain is more common among children and teenagers of developing countries [8–10]. For example, in Iran, neck or shoulder pain was reported 28.6% among 11–14-year-old children [11]. Lifestyle, physical factors, psychological factors, and social factors and improper sitting have been identified as the risk factors associated with neck and shoulder pain among students in different studies [3, 12–17]. Furthermore, neck and shoulder pain among children is considered as a risk factor for health problems during adulthood [18, 19]. Therefore, detecting and understanding the pain and managing it during childhood and adolescence is needed to prevent such problems. Given the importance of healthier body composition in childhood which is closely related to healthier profile in adulthood later, it is absolutely essential to identify the risk factors which are major contributors to health problems among children [20]. However, the results on the risk factors (e.g. physical and leisure activity, psychosocial variables, and school-related factors such as classroom furniture) and their relationship to neck and shoulder pain among children and teenagers are not consistent in different studies [21–25]. In additon, the number of the studies which consider all the above mentioned factors is limited. Moreover, little research has focused on the prevalence and the risk factors associated with shoulder and neck pain among the students in elementary schools [9, 11]. Therefore, this study seeks to assess the prevalence of shoulder and neck pain and its potential risk factors among elementary school students.

## Methods

**Fig. 1:**
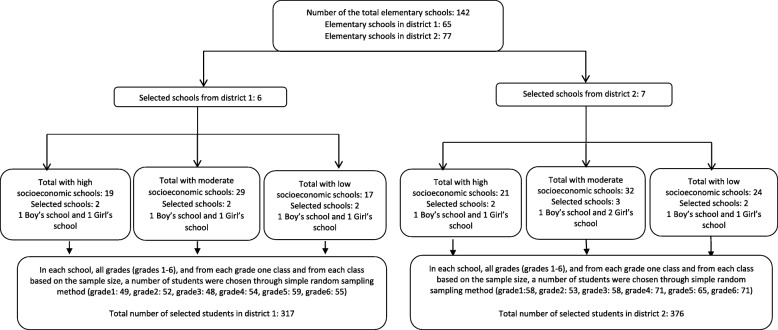
Flow chart of sampling

The participants in this cross-sectional study were 693 elementary school students (318 boys and 375 girls), and their parents. They were all from Hamadan, a city located in the west of Iran. Students were in grades 1–6 (7 to 12 years old). Data was collected from 20 January to 17 March 2018. The sample size was estimated by using th*e* $\kern0.5em n=\kern0.5em \frac{{\left({\approx}_{a/2}\right)}^2p\left(1-p\right)}{E^2}$ formula, taking into account the 95% confidence level, 0.95 (1-α = 0.95), the same prevalence that Dianat et al. found in their study [11], with a *p* = 0.28 and an estimation error (E) of maximum 15% p and applying a cluster sampling factor of 1.5. Therefore the sample size was estimated as 670 students. A random sampling method with a multistage design was used in this study. Hamadan has two educational districts which were considered for sampling. At the first step, the list of elementary schools was prepared and then schools from districts 1 and 2 with high, moderate and low socioeconomic statuses were chosen. Accordingly, 13 schools from the mentioned districts (six from district 1 and 7 from district 2) in terms of gender classifications (male/female) were chosen, as schools in Iran are not coeducational, girls and boys study in single-sex schools. In each school, one classroom from each grade, and then from each classroom, a number of students were chosen through the simple random sampling method based on the sample size (Fig. 1). Finally, the students were selected according to the following criteria for inclusion: (1) being a student in elementary school, and (2) not having chronic diseases or musculoskeletal constraints. The exclusion criteria included not having consent to participate in the study (students and/or their parents).

Prior to collecting data, permissions were received from Education office authorities to collect data in the schools, and the students were informed that the participation in the study was voluntary. Oral informed consents from the students and the written informed consents from students’ parents were obtained. There were 780 eligible individuals, of which 693 participated in the study (participation rate = 88.8%).

For the questions about demographic variables, physical activity / leisure time and school related variables, Dianat et al. and Hatami et al., questionnaires (in Persian) were used. These questionnaires have been shown to be adequately reliable and valid [11, 26]. Minor revisions were made according to the experts’ opinions to adapt the items of the questionnaires for the study population in the present study. The questionnaires were tested in a pilot study on 60 students. According to their comments, partial revisions were made to a number of items in the questionnaire to improve transparency and understandability. Additionally, the test-retest approach was used to assess the reliability of the questionnaire items using the Kappa Coefficient (ranging from 0.73 to 0.95) and Internal Correlation Coefficient (ranging from 0.85 to 0.98). The questionnaire developed consisted of three parts. The first part was about the demographic variables and physical activity/leisure time (including age, gender, school grade, physical activity, using of cell phone, using of computer, computer games, and watching television). The second part focused on the school associated factors and information about the suitability and comfort of the school furniture (based on a modification of the Chair Feature Checklist), and the design of the classroom and amount of homework. The last part of the questionnaire included questions about the type of school backpack, how students carry backpack to school and the duration of backpack carrying to school.

To evaluate the students’ posture, the Rapid Upper Limbs Assessment (RULA) checklist was used. RULA which had been developed as an observational method for investigating improper postures among workers was used in this study. First, observations were recorded as numerical scores, then these scores were converted to the final scores through the RULA specific matrix scoring, so that higher scores represented improper postures. The classification of scores was as follow: Level 1: a score of one or two which indicates that the posture is acceptable if not maintained for a long period of time. Level 2: a score of three or four which indicates that further investigation is needed and changes may be required. Level 3: a score of five or six which indicates that the investigation is needed and changes are required, soon. Level 4: a score of seven or more which indicates that further investigation and immediate changes are required [27]. The validity of the RULA checklist in Iran was previously examined by Dianat and Salimi [28] whereas, its reliability was assessed, through inter-rater reliability, at two different times, using a pilot study on 60 students in the current study. The Cronbach’s alpha coefficients for upper arm, lower arm, wrist, neck, trunk, and leg were reported as 0.79, 0.82, 0.78, 0.83, 0.84, and 0.86, respectively.

The standard Strengths and Difficulties Questionnaire (SDQ), was used to measure psychosocial factors [29], and filled out by the parents. The SDQ is a behavioral screening questionnaire with 25 items measuring five behavioral and emotional dimensions in children under the subscales of hyperactivity, emotional problems, conduct problems, peer problems, and prosocial behaviors. The score of prosocial behaviors represented the score of strengths, while the sum of score of other items constituted the score of difficulties. The parents were requested to choose from “not true”, “somewhat true”, and “certainly true” to answer each question, with a range of scores from 0 to 2. Every subscale consisted of five questions and the range of score of strengths was 0–10 and for that of difficulties was 0–40. Given the score, each subscale was divided into three classes of normal, borderline, and abnormal. Reliability and validity of the questionnaire in Iran has been confirmed by Tehranidost et al. [30].

The prevalence of neck and shoulder pain was evaluated by preshaded manikin pictures in order to show the desired areas. The question was as follows “ Have you ever experienced any pain or discomfort in desired areas for one day or more over the past month?” followed by the response (yes/no) [11, 23]. Regarding the severity of pain, the Visual Analog Scale (VAS) was used with a range of 0–10 with 0 representing no pain and 10 indicating maximum pain [31].

Finally, the students’ weight and their backpacks weight were measured using an electronic digital scale (Beurer, Germany) with a 100 g accuracy. The students’ height was measured in centimeters and using a portable stadiometer. Body Mass Index (BMI) was calculated for each student (kg/m2) and classified according to the WHO growth references (2007) into three categories: underweight (<5th percentile), healthy weight (≥5th to <85th percentiles), extra weight (including overweight and obese) (≥85th percentile) [32].

All questionnaires were completed through interviewing and by the principal investigator. The students’ posture was examined by an observer specialized in ergonomics.

Data was analyzed via the SPSS software v.23. The relationship between the shoulder and neck pain, and the study variables was analyzed by the unadjusted (univariate) logistic regression, and those factors independently associated with the shoulder and neck pain were assessed using the adjusted (multivariate) logistic regression. The effect size was calculated using the chance ratio with a confidence interval of 95%, and a significance level less than 0.05.

## Results

A total of 32.3% (224) students had at least one of the neck and shoulder pain problems, and 17.2% (119 individuals) reported pain in both regions. The presence of neck and shoulder pain was 35.8% (*n* = 248) and 30.9% (*n* = 214), respectively. Further, the mean and standard deviation of severity of pain in neck and shoulder areas were reported as 1.36 ± 0.48 and 1.31 ± 0.46, respectively (with a range of 0–10).

### Demographic factors and physical activity/leisure time

The mean (±standard deviation) of the demographic variables among students were 9.70 (±1.61) years for the age, 35.88 (±16.85) kg for the weight, 136 (±16.32) cm for the height, 3.69 (±0.74) kg for the backpack weight and 17.68 (±3.57) kg/m2 for the BMI.

According to Table 1, the boys reported significantly less shoulder pain than the girls (OR = 0.69, 95% CI: 0.50–0.96; *p* < 0.030). Also the use of cell phone/tablet for 1–3 h per day was significantly associated with neck pain (OR = 2.11, 95% CI: 1.08–4.13; *p* < 0.029).

**Table 1:** Demographic and Physical/leisure activity factors and risk of neck and shoulder pain in unadjusted (univariate) logistic regression analysis

Neck pain				Shoulder pain		
	%	OR (95% CI)	*P* value	%	OR (95% CI)	*P* value
Age (year)						
< 10	38.2	Referent	–	29.1	Referent	–
≥ 10	33.9	1.20 (0.88–1.64)	0.237	32.3	0.86 (0.62–1.93)	0.370
Gender						
Boys	33.3	Referent	–	26.7	Referent	–
Girls	37.9	0.82 (0.60–1.12)	0.215	34.3	0.69 (0.50–0.96)	0.030*
BMI (kg/m2)						
healthy weight (≥5th to <85th percentile)	36.2	Referent	–	28.9	Referent	–
under weight (<5th percentile)	31.9	0.82 (0.43–1.57)	0.561	31.9	1.15 (0.60–2.19)	0.666
extra weight (≥85th percentile)	35.8	0.98 (0.68–1.40)	0.918	35.8	1.36 (0.95–1.97)	0.092
Hours per week playing sport (h)						
< 1	39.7	Referent	–	27.2	Referent	–
1–3	34.9	1.30 (0.77–2.18)	0.342	32	0.80 (0.46–1.38)	0.429
> 3	33.7	1.05 (0.67–1.66)	0.806	31.7	1.01 (0.63–1.60)	0.963
Hours per day using a cell phone/tablet (h)						
< 1	22.1	Referent	–	30.9	Referent	–
1–3	35.4	2.11 (1.08–4.13)	0.029*	28.6	0.70 (0.39–1.26)	0.239
> 3	37.6	1.92 (0.95–3.87)	0.068	38.9	2.12 (0.33–1.17)	0.148
Hours per day using a computer (h)						
< 1	36	Referent	–	31	Referent	–
1–3	35.6	1.40 (0.43–4.52)	0.570	28.8	0.80 (0.26–2.44)	0.705
> 3	28.6	1.38 (0.38–4.95)	0.620	35.7	0.72 (0.23–2.49)	0.614
Hours per day playing games (h)-						
< 1	35.9	Referent	0.305	30.7	Referent	0.761
1–3	38.8	1.54 (0.67–3.52)	0.250	31.3	0.88 (0.40–1.93)	0.846
> 3	26.7	1.74 (0.67–4.49)		33.3	0.91 (0.36–2.28)	
Hours per day watching TV (h)						
< 1	38.8	Referent	–	29.1	Referent	–
1–3	35	1.22 (0.82–1.80)	0.320	31.3	0.86 (0.57–1.30)	0.499
> 3	34.2	1.03 (0.71–1.50)	0.843	32.7	0.95 (0.65–1.39)	0.819

*Statistically significant, −: Not available, *CI* Confidence interval, *%* Relative frequency, *OR* Odds Ratio

### Factors associated with school and curricula

Table 2 shows the relationship between school-associated factors, and neck and shoulder pain. Factors associated with neck pain in the unadjusted (univariate) analysis were very high, seat height (OR = 2.76, 95% CI: 1.78–4.29; *p* < 0.001), very short seat height (OR = 2.34, 95% CI: 1.35–4.06; *p* < 0.002), very high desk height (OR = 2.64, 95% CI: 1.61–4.32; *p* < 0.001), very short desk height (OR = 1.96, 95% CI: 1.30–2.96; *p* < 0.001), too backward seat pan inclination (OR = 1.51, 95% CI: 1.03–2.23; *p* < 0.033), too forward seat pan inclination (OR = 2.04, 95% CI: 1.05–3.98; *p* < 0.035), difficulty in viewing the classroom board (OR = 2.34, 95% CI: 1.08–5.04; *p* < 0.030) and too much homework (OR = 2.19, 95% CI: 1.32–3.62; *p* < 0.002). The students who did their homework on desk and seats (OR = 0.63, 95% CI: 0.43–0.92; *p* < 0.018) and laying position at home (OR = 0.54, 95% CI: 0.37–0.79; *p* < 0.002) reported less neck pain.

**Table 2:** School-related factors and risk of neck and shoulder pain in unadjusted (univariate) logistic regression analysis

Neck pain				Shoulder pain		
	%	OR (95% CI)	*P* value	%	OR (95% CI)	*P* value
Classroom furniture/layout design						
Seat height						
Just right	20.1	Referent	–	24	Referent	–
Too high	37.2	2.76 (1.78–4.29)	0.001*	31.9	1.56 (1.02–2.38)	0.037*
Too low	41.1	2.34 (1.35–4.06)	0.002*	33.1	1.47 (0.86–2.54)	0.157
Seat backrest height						
Just right	35.1	Referent	–	29.4	Referent	–
Too high	36.4	1.05 (0.74–1.49)	0.753	29.8	0.84 (0.59–1.20)	0.352
Too low	35	1.06 (0.67–1.66)	0.792	33.1	0.85 (0.53–1.36)	0.516
Seat backrest inclination						
Just right	36.4	Referent	–	19.4	Referent	–
Too backward	37.8	1.53 (0.94–2.49)	0.084	31.7	2.07 (1.20–3.55)	0.008*
Too forward	27.6	1.59 (0.93–2.72)	0.087	33.3	1.92 (1.06–3.48)	0.030*
Seat backrest curvature						
Just right	36.7	Referent	–	27.2	Referent	–
Too curved	41	1.12 (0.80–1.57)	0.502	34.6	1.44 (1.01–2.04)	0.039*
Too flat	34.1	1.34 (0.81–2.21)	0.243	35.1	1.41 (0.84–2.37)	0.192
Seat depth						
Just right	34.7	Referent	–	30.7	Referent	–
Too deep	43.5	0.97 (0.70–1.35)	0.892	34.8	1.02 (0.72–1.43)	0.909
Too narrow	35.2	1.41 (0.83–2.40)	0.196	30.2	1.23 (0.71–2.13)	0.460
Seat width						
Just right	35.5	Referent	–	31.1	Referent	–
Too deep	31	0.86 (0.58–1.25)	0.437	25.4	0.92 (0.62–1.36)	0.685
Too narrow	39	0.70 (0.38–1.28)	0.248	32.9	0.69 (0.36–1.36)	0.260
Desk height						
Just right	23.7	Referent	–	21.9	Referent	–
Too high	37.9	2.64 (1.61–4.32)	0.001*	41.2	1.62 (1.06–2.47)	0.024*
Too low	45	1.96 (1.30–2.96)	0.001*	31.3	2.50 (1.51–4.14)	0.001*
Seat pan inclination						
Just right	28.3	Referent	–	31	Referent	–
Too backward	44.7	1.51 (1.03–2.23)	0.033*	31.9	1.04 (0.71–1.53)	0.825
Too forward	37.5	2.04 (1.05–3.98)	0.035*	30.1	1.08 (0.54–2.18)	0.814
Seat-to-(black) board distance						
Just right	33.7	Referent	–	30.1	Referent	–
Too near	39.4	0.98 (0.58–1.66)	0.958	30.3	086 (0.49–1.49)	0.592
Too far	39.7	0.77 (0.52–1.13)	0.186	33.6	0.85 (0.57–1.27)	0.437
Classroom teacher placement						
Just right	37.6	Referent	–	32.6	Referent	–
Too near	29.9	0.95 (0.49–1.85)	0.890	26.9	1.08 (0.54–1.17)	0.811
Too far	30.8	1.35 (0.86–2.11)	0.189	25.2	1.43 (0.89–2.29)	0.138
Viewing the (black) board						
Very easy	24.5	Referent	–	31.9	Referent	–
Neutral	43.2	1.69 (0.86–3.33)	0.124	27.4	1.29 (0.67–2.50)	0.441
Very difficult	35.5	2.34 (1.08–5.04)	0.030*	26.5	1.04 (0.47–2.27)	0.951
Hear the teacher’s voice						
Very easy	36.1	Referent	–	31	Referent	–
Neutral	30.8	0.79 (0.24–2.52)	0.691	32.7	2.24 (0.48–1.35)	0.299
Very difficult	41.7	0.62 (0.17–2.60)	0.471	16.7	2.42 (0.47–1.33)	0.285
Viewing the book/notebook						
Very easy	35.5	Referent	–	30.5	Referent	–
Neutral	37.9	0.68 (018–2.58)	0.578	32.8	0.54 (0.14–2.06)	0.225
Very difficult	44.4	0.76 (0.18–3.15)	0.710	44.4	0.60 (0.14–2.53)	0.365
Homework						
Just right	26.7	Referent	–	22.2	Referent	–
Not enough	18.8	0.63 (0.34–1.16)	0.145	16.5	0.69 (0.36–1.31)	0.257
Too much	44.3	2.19 (1.32–3.62)	0.002*	38.3	2.17 (1.27–3.70)	0.004*
Position doing homework at home						
Sitting on the floor	33.3	Referent	–	32.5	Referent	–
Sitting on the table and the chair	30	0.63 (0.43–0.92)	0.018*	29.6	1.09 (0.73–1.61)	0.661
Lying on the floor	44.1	0.54 (0.37–0.79)	0.002*	30.6	0.95 (0.64–1.42)	0.823
Schoolbag carriage variables						
Type of school bag						
Backpack	35.9	Referent	–	31.4	Referent	–
Other (shoulder bag/wheeled)	29.4	1.34 (0.46–3.86)	0.580	11.8	3.42 (0.77–15.11)	0.104
School bag weight as % BW						
≤ 10	33.5	Referent	–	30.4	Referent	–
> 10	37.2	0.84 (0.61–1.17)	0.318	31.2	0.96 (0.69–1.34)	0.837
Time spent carrying school bag (min/day)						
≥ 20	36.3	Referent	–	29.2	Referent	–
> 20	33.8	11.1 (0.74–1.65)	0.602	36.8	0.71 (0.48–1.06)	0.099
Method of school bag carriage						
Both shoulders	34.2	Referent	–	30.6	Referent	–
One shoulders	44.2	1.06 (0.39–2.86)	0.909	38.4	1.41 (0.88–2.26)	0.147
Other (by hands/wheels)	33.3	1.58 (0.54–4.60)	0.399	5.6	0.13 (0.01–1.01)	0.051
Method of travel to/from school						
Car	33	Referent	–	30.3	Referent	–
Walk	35.4	0.58 (0.32–0.95)	0.032*	29.7	0.71 (0.43–1.20)	0.207
Other	45.9	0.64 (0.40–1.04)	0.076	37.6	0.69 (0.42–1.14)	0.157

*Statistically significant, −: Not available, *CI* Confidence interval, *%* Relative frequency, *OR* Odds Ratio

Unadjusted (univariate) analysis indicated that shoulder pain was significantly associated with very high seat height (OR = 1.56, 95% CI: 1.02–2.38; *p* < 0.037), too backward seat backrest inclination (OR = 2.07, 95% CI: 1.20–3.55; *P* < 0.008), too forward seat backrest inclination (OR = 1.92, 95% CI: 1.06–3.48; *p* < 0.030), too curved seat backrest curvature (OR = 1.44, 95% CI: 1.01–2.04; *p* < 0.039), very high desk height (OR = 1.62, 95% CI: 1.06–2.47; *p* < 0.024), very short desk height (OR = 2.50, 95% CI: 1.51–4.14; *p* < 0.001) and too much homework (OR = 2.17, 95% CI: 1.27–3.70; *p* < 0.004).

### Psychosocial factors

Table 3 presents the relationship between psychosocial factors and prevalence of neck and shoulder pain. No statistically significant difference was found between neck pain and psychosocial factors. However, only borderline peers’ problems (OR = 1.56, 95% CI: 1.02–2.40; *p* < 0.039) had a significant relationship with shoulder pain.

**Table 3:** Psychosocial factors and risk of neck and shoulder pain in unadjusted (univariate) logistic regression analysis

Neck pain				Shoulder pain		
	%	OR (95% CI)	*P* value	%	OR (95% CI)	*P* value
Strengths						
Prosocial behavior						
Normal	36.2	Referent		30.5	Referent	
Borderline	20.8	0.46 (0.17–1.25)	0.132	41.7	1.62 (0.71–3.72)	0.251
Abnormal	42.9	1.32 (0.45–3.85)	0.609	28.6	0.91 (0.28–2.93)	0.875
Difficulties						
Emotional symptoms						
Normal	34.4	Referent		30.9	Referent	
Borderline	38.6	1.20 (0.68–2.10)	0.525	29.8	0.95 (0.52–1.72)	0.871
Abnormal	40.5	1.30 (0.86–1.95)	0.206	31.4	1.02 (0.66–1.57)	0.909
Conduct problems						
Normal	33.7	Referent		30.4	Referent	
Borderline	40.6	1.34 (0.86–2.11)	0.193	35.4	1.25 (0.79–1.99)	0.329
Abnormal	40.5	1.34 (0.88–2.03)	0.167	29.3	0.95 (0.61–1.48)	0.826
Hyperactivity						
Normal	35.2	Referent		30.6	Referent	
Borderline	37.1	1.08 (0.64–1.82)	0.747	34.3	1.18 (0.69–2.00)	0.531
Abnormal	38	1.12 (0.72–1.75)	0.590	30.3	0.97 (0.61–1.55)	0.906
Peer problems						
Normal	34.2	Referent		23.7	Referent	
Borderline	33.3	1.14 (0.77–1.69)	0.504	33.3	1.56 (1.02–2.40)	0.039*
Abnormal	37.3	0.96 (0.59–1.55)	0.873	32.7	1.61 (0.96–2.68)	0.067
Total difficulties						
Normal	31.7	Referent		30.8	Referent	
Borderline	24.6	1.28 (0.82–1.99)	0.274	32.3	1.07 (0.67–1.70)	0.758
Abnormal	48.4	1.10 (0.71–1.71)	0.661	30.1	0.96 (0.61–1.54)	0.969

*Statistically significant, −: Not available, *CI* Confidence interval, *%* Relative frequency, *OR* Odds Ratio

### RULA score and prevalence of neck and shoulder pain

Table 4 shows the relationship between RULA score and prevalence of neck and shoulder pain. RULA score level III (OR = 2.15, 95% CI: 1.29–3.61; *p* < 0.003), RULA score level IV (OR = 2.64, 95% CI: 1.53–4.56; *p* < 0.001), and neck pain, as well as RULA at level III (OR = 1.72, 95% CI: 1.02–2.89; *p* < 0.039), and RULA at level IV (OR = 1.83, 95% CI: 1.06–3.18; *p* < 0.030) had statistically significant relationships with shoulder pain.

**Table 4:** RULA score and risk of neck and shoulder pain in unadjusted (univariate) logistic regression analysis

Neck pain				Shoulder pain		
	%	OR (95% CI)	*P* value	%	OR (95% CI)	*P* value
RULA						
Level 1	16.1	Referent		14.3	Referent	
Level 2	40.9	0.59 (0.30–1.18)	0.140	35.5	0.52 (0.25–1.05)	0.068
Level 3	45.9	2.15 (1.29–3.61)	0.003*	37	1.72 (1.02–2.89)	0.039*
Level 4	24.2	2.64 (1.53–4.56)	0.001*	24.2	1.83 (1.06–3.18)	0.030*

*Statistically significant, −: Not available, *CI* Confidence interval, *%* Relative frequency, *OR* Odds Ratio

According to the adjusted (multivariate) regression analysis (Table 5), factors with direct association with neck pain were very high desk height (OR = 1.96, 95% CI: 1.02–3.74; *p* < 0.001), backward seat pan inclination (OR = 2.10, 95% CI: 1.37–3.24; p < 0.001), forward seat pan inclination (OR = 3.12, 95% CI: 1.46–6.68; *P* < 0.003), difficulty in viewing the classroom board (OR = 2.54, 95% CI: 1.10–5.84; *p* < 0.028), too much homework (OR = 2.59, 95% CI: 1.49–4.51; *p* < 0.001), RULA score level III (OR = 2.88, 95% CI: 1.64–5.05; p < 0.001) and RULA score level IV (OR = 3.12, 95% CI: 1.72–5.63; *p* < 0.001). The students who did performed their homework on desk and (OR = 0.59, 95% CI: 0.39–0.91; *p* < 0.017) and in a laying position (OR = 0.53, 95% CI: 0.34–0.81; *p* < 0.004) reported less pain.

**Table 5:** Factors associated with neck pain in adjusted (multivariate) logistic regression analysis

Neck pain		
	R (95% CI)	*P* value
School-related factors		
Desk height		
Just right	Referent	
Too high	1.96 (1.02–3.74)	0.042*
Too low	1.01 (0.58–1.73)	0.976
Seat pan inclination		
Just right	Referent	
Too backward	2.10 (1.37–3.24)	0.001*
Too forward	3.12 (1.46–6.68)	0.003*
Viewing the (black) board		
Very easy	Referent	
Neutral	1.87 (0.96–3.63)	0.062
Very difficult	2.54 (1.10–5.84)	0.028*
Homework		
Just right	Referent	
Not enough	0.89 (0.46–1.72)	0.740
Too much	2.59 (1.49–4.51)	0.001*
Position doing homework at home		
Sitting on the floor	Referent	
Sitting on the table and the chair	0.59 (0.39–0.91)	0.017*
Lying on the floor	0.53 (0.34–0.81)	0.004*
RULA		
Level 1	Referent	
Level 2	0.84 (0.40–1.75)	0.655
Level 3	2.88 (1.64–5.05)	0.001*
Level 4	3.12 (1.72–5.63)	0.001*

*Statistically significant, *CI* Confidence interval, *OR* Odds Ratio

According to adjusted (multivariate) regression analysis (Table 6), factors independently related to shoulder pain were very short desk height (OR = 2.41, 95% CI: 1.26–4.61; *p* < 0.008) and too much homework (OR = 1.94, 95% CI: 1.10–3.42; *p* < 0.022).

**Table 6:** Factors associated with shoulder pain in adjusted (multivariate) logistic regression analysis

Shoulder pain		
	OR (95% CI)	*P* value
School-related factors		
Desk height		
Just right	Referent	
Too high	1.21 (0.70–2.07)	0.481
Too low	2.41 (1.26–4.61)	0.008*
Homework		
Just right	Referent	
Not enough	0.82 (0.42–1.62)	0.579
Too much	1.94 (1.10–3.42)	0.022*

*Statistically significant, *CI* Confidence interval, *OR* Odds Ratio

## Discussion

This study investigated the prevalence of neck and shoulder pain among 7–12 years old students. More than one-third of students in this study had at least one of shoulder and neck pain problems. Furthermore, physical factors such as school furniture, too much homework, difficulty in viewing the board, and posture (improper sitting positions) were associated with the pain.

Neck pain was associated with very high desk height, as well as forward and backward seat pan inclination. On the other hand, shoulder pain was associated with very short desk height.

Improper height of desk and seat often led to abnormal postures as well as prevalence of shoulder and neck pain [10]. High desk height compels students to lift their arms, causing heavier muscular loads, which in turn results in pain and discomfort in shoulder and neck [33]. Short desk height encourages the back part of the body to lean to a forward position, and thus under load, which could be a possible contributing factor to the pain [34, 35]. Two studies performed in Hamadan (a city in Iran) confirm the mismatch between the anthropometric dimensions of the students and the furniture. The studies found that, the use of same sized furniture in the elementary schools of Hamadan for all educational grades has increased the mismatch with the students’ anthropometric dimensions [36, 37]. Therefore, ergonomic interventions with the aim of improving physical factors in the school environment (designing suitable furniture) may help preventing of such complaints [38–41].

In the present study, the amount of homework was significantly associated with shoulder and neck pain, that is, the students who spent a lot of time on doing homework had more shoulder and neck pains. This finding was in line with the findings of the study by Dianat et al. [11]. However, the students who did their homework on desk and seat or in a laying position reported less pain. It is believed that the worst method of doing homework is bending on the floor [42]. In this position of studying, the spinal cord is bent for a long time which causes extension and muscle weakness in the spinal cord. Furthermore, with a reduction of abductor muscles of the upper part of the body and shoulder due to different reasons in sitting and standing positions for a long time, the extent of kyphosis curve increases [43, 44]. The abductor muscles are needed to produce the force required to hold the vertebral column straight. By decreasing the ability of these muscles to generate force, the spinal column does not have sufficient support for the abductor muscles to keep it straight in standing and sitting postures. As a result, the weight and force of the upper part of the trunk are placed on the inactive limbs of the ligaments, bones and articular cartilage, etc. This weight can increase the length of the abductor muscles, thus affecting the arches of the vertebral column and increasing the kyphosis arc [45]. Doing homework in a bending position on the floor increases the percentage of kyphosis, but the use of desk decreases it [42, 44].

Difficulty in viewing the board was one of the physical factors associated with neck pain, which is consistent with the results of Dianat et al’s study [11].. It might be attributed to the improper arrangement of desks and seats, unsuitable distance of the blackboard, improper lighting of the class, and other ergonomic principles in the classroom.

RULA score levels III and IV were associated with neck pain in the current study. This supports the findings of a number of previous studies that have reported a significant relationship between bending and rotating of neck and musculoskeletal pains of neck in school age children and adolescents. However, these studies used different instruments (Portable Ergonomic Observation Method/sagittal plane digital photographs) [46–48]. Students usually sit with improper postures as bending their neck, trunk, and back for a long time or rotating them, which in turn causes musculoskeletal pain [47–49]. The poor postures and prolonged sitting are common in the classroom [50, 51]. In the present study, given the RULA score at levels three and four (investigation and changes are required soon and immediately), education regarding the proper ways of positioning the head and neck to prevent these complaints is recommended [20, 50, 52].

Individual factors such as physical/leisure activity (physical activity time, using cellphone and computer, playing games, watching TV) and BMI were not significantly related to neck and shoulder pain which is in line with other studies conducted by Diepenmaat et al. in Netherland [23] Murphy et al. in England [34] and Dianat et al. in Iran [11].

This study indicated that psychosocial factors in the adjusted (multivariate) regression had no significant relationship with shoulder and neck pain. This stands contrary to the previous research which has reported the relationship between psychosocial factors and musculoskeletal complaints in school age children and adolescents [8, 16, 53, 54]. This may result from the social acceptance of the self-report data. Concerning the young age of students, the SDQ questionnaire was completed by their parents as a self-report. By considering the importance of children’s health for their parents and also their tendency to report their children’s psychosocial conditions desirably, these can have an impact on the study findings.

The response rate in this study was high. Due to the probability of incomplete ability of students to responding the questions, the interview, as the data gathering strategy, was used in this study. Furthermore, to enhance the accuracy of data, posture of students was observed and evaluated by a specialist observer.

Despite the robust findings, this study has several limitations. This study had a cross-sectional design and the cause-effect relationship between shoulder and neck pain and its risk factors could not be established. Another limitation was using self-report manner for shoulder and neck pain reported by children and their parents. Although other studies also used this procedure of data collection [55, 56], the use of a subjective measurement without a physical examination is not sufficient for the assessment of prevalence of neck and shoulder pain. This is the limitation of RULA checklist that does not consider angles of side bending and twisting and also only takes one posture into account for those students who have a high amount of displacement in class.

## Conclusion

The prevalence of neck and shoulder pain was high in the studied population. Unsuitability of school furniture, too much homework, difficulty in viewing the classroom board, and improper sitting positions of students are related to this health problem. Therefore, the proper interventions, considering the risk factors assessed in this study are suggested.

## Data Availability

The datasets used and analyzed during the current study are available from the corresponding author on reasonable request.

## Abbreviations

BMI: Body Mass Index
CI: Confidence interval
OR: Odds ratio
RULA: Rapid upper limbs assessment
SDQ: Strengths and difficulties of questionnaire
